# Feeding a Protective Hydrolysed Casein Diet to Young Diabetes-prone BB Rats Affects Oxidation of L[U−C14] glutamine in Islets and Peyer's Patches, Reduces Abnormally High Mitotic Activity in Mesenteric Lymph Nodes, Enhances Islet Insulin and Tends to Normalize NO Production

**DOI:** 10.1155/EDR.2000.121

**Published:** 2000

**Authors:** Willy J. Malaisse, Elizabeth Olivares, Aouatif Laghmich, Laurence Ladrière, Abdullah Sener, Fraser W. Scott

**Affiliations:** 1 Laboratory of Experimental Medicine Brussels Free University Brussels Belgium; 2 Ottawa Hospital Research Institute The Ottawa Hospital-General Campus Laboratory N1 501 Smyth Road Ottawa Ontario K1H 8L6 Canada

## Abstract

The present studies were undertaken to examine concomitant diet-induced changes in pancreatic islets and cells of the gut immune system of diabetes-prone BB rats in the period before classic insulitis. Diabetes-prone (BBdp) and control non-diabetes prone (BBc) BB rats were fed for ~ 17 days either a mainly plant-based standard laboratory rodent diet associated with high diabetes frequency, NIH-07 (NIH) or a protective semipurified diet with hydrolyzed casein (HC) as the amino acid source. By about 7 weeks of age, NIH-fed BBdp rats had lower plasma insulin and insulin/glucose ratio, lower insulin content of isolated islets, lower basal levels of NO but higher responsiveness of NO production to IL-1*β* in cultured islets, and higher Con A response and biosynthetic activities in mesenteric lymphocytes than control rats fed the same diet. In control rats, the HC diet caused only minor changes in most variables, except for a decrease in oxidation of L-[U−C14]glutamine in Peyer's patch (PP) cells and an increase in protein biosynthesis in mesenteric lymphocytes. In BBdp rats, however, the HC diet increased plasma insulin concentration, islet insulin/ protein ratio, and tended to normalize the basal and IL-1*β*-stimulated NO production by cultured islets. The HC diet decreased oxidation of L-[U−C14]glutamine in BBdp pancreatic islets, whereas oxidation of L-[U−C14]glutamine in PP cells was increased, and the basal [Methyl-H3] thymidine incorporation in mesenteric lymphocytes was decreased. These findings are compatible with the view that alteration of nutrient catabolism in islet cells as well as key cells of the gut immune system, particularly changes in mitotic and biosynthetic activities in mesenteric lymphocytes, as well as basal and IL-1*β* stimulated NO production, participate in the sequence of events leading to autoimmune diabetes in BB rats. Thus, the protection afforded by feeding a hydrolysed casein-based diet derives from alterations in both the target islet tissue and key cells of the gut immune system in this animal model of type 1 diabetes.


*Int. Jnl. Experimental Diab. Res.*, Vol. 1, pp. 121–130
Reprints available directly from the publisher
Photocopying permitted by license only

© 2000 OPA (Overseas Publishers Association) N.V.
Published by license under
the Harwood Academic Publishers imprint,
part of The Gordon and Breach Publishing Group.
Printed in Malaysia.

### Feeding a Protective Hydrolysed Casein Diet to Young Diabetes-prone BB Rats Affects Oxidation of L[U-$^{14}$C]glutamine in Islets and Peyer's Patches, Reduces Abnormally High Mitotic Activity in Mesenteric Lymph Nodes, Enhances Islet Insulin and Tends to Normalize NO Production

WILLY J. MALAISSE[a], ELIZABETH OLIVARES[a], AOUATIF LAGHMICH[a], LAURENCE LADRIÈRE[a], ABDULLAH SENER[a] and FRASER W. SCOTT[b,*]

[a] *Laboratory of Experimental Medicine, Brussels Free University, Brussels, Belgium;* [b] *Ottawa Hospital Research Institute, The Ottawa Hospital-General Campus, Laboratory N1, 501 Smyth Road, Ottawa, Ontario, K1H 8L6, Canada*

*(Received 1 June 1999; In final form 19 January 2000)*

**The present studies were undertaken to examine concomitant diet-induced changes in pancreatic islets and cells of the gut immune system of diabetes-prone BB rats in the period before classic insulitis. Diabetes-prone (BBdp) and control non-diabetes prone (BBc) BB rats were fed for ~17 days either a mainly plant-based standard laboratory rodent diet associated with high diabetes frequency, NIH-07 (NIH) or a protective semipurified diet with hydrolyzed casein (HC) as the amino acid source. By about 7 weeks of age, NIH-fed BBdp rats had lower plasma insulin and insulin/glucose ratio, lower insulin content of isolated islets, lower basal levels of NO but higher responsiveness of NO production to IL-1$\beta$ in cultured islets, and higher Con A response and biosynthetic activities in mesenteric lymphocytes than control rats fed the same diet. In control rats, the HC diet caused only minor changes in most variables, except for a decrease in oxidation of L-[U-$^{14}$C]glutamine in Peyer's patch (PP) cells and an increase in protein biosynthesis in mesenteric lymphocytes. In BBdp rats, however, the HC diet increased plasma insulin concentration, islet insulin/protein ratio, and tended to normalize the basal and IL-1$\beta$-stimulated NO production by cultured islets. The HC diet decreased oxidation of L-[U-$^{14}$C]glutamine in BBdp pancreatic islets, whereas oxidation of L-[U-$^{14}$C]glutamine in PP cells was increased, and the basal [Methyl-$^{3}$H]thymidine incorporation in mesenteric lymphocytes was decreased. These findings are compatible with the view that alteration of nutrient catabolism in islet cells as well as key cells of the gut immune system, particularly changes in mitotic and biosynthetic activities in mesenteric lymphocytes, as well as basal and IL-1$\beta$ stimulated NO production, participate in the sequence of events leading to autoimmune diabetes in BB rats. Thus, the protection afforded by feeding a hydrolysed casein-based diet derives from alterations in both the target islet tissue and key cells of the gut immune system in this animal model of type 1 diabetes.**

*Corresponding author. Tel.: 613-737-8929, Fax: 613-739-6189, e-mail: fscott@ogh.on.ca

*Keywords:* BB rat, type 1 diabetes, diet, autoimmunity, gut, IL-1$\beta$

*Abbreviations:* BB, BioBreeding rat; BBdp, diabetes-prone BB rat; BBc, control (non-diabetes-prone) BB rat; NO, nitric oxide; NIH, NIH-07, mainly plant-based (diabetes-promoting) diet; HC, hydrolysed casein-based (low diabetes) semipurified AIN-93G diet; PP, Peyer's patch; MLN, mesenteric lymph node; IL-1$\beta$, interleukin-1$\beta$

### INTRODUCTION

Certain diets affect the development of spontaneous, autoimmune type 1 diabetes in susceptible BB rats,[1] NOD mice[2,3] and possibly humans.[4,5] The identity of some foods that can induce diabetes in BB rats is known[6] and feeding susceptible rats a protective diet such as a hydrolysed casein (HC)-based semipurified mixture inhibits the appearance of diabetes and dampens the severity of pancreatic islet inflammation.[1,6–8] In BBdp rats fed the HC diet from weaning, pancreatic islet cell class I MHC expression was low,[7] a more normal islet mass was seen early in life and the smaller numbers of cells that infiltrated the pancreas and islets at around 70 days of age were predominantly Th2 or Th3 cells, more characteristic of a benign insulitis.[8,9] The fact that food is an important determinant of diabetes outcome in BB rats and NOD mice, suggests that it may be possible to modify diabetogenesis by targeting the gut immune system.[10,11] However, there is scant information on concomitant changes in cells of the gut and pancreas in animals fed sources of dietary amino acids that have different diabetes-inducing capacity. It was reported that feeding an HC diet for $\sim$20 days to BBdp rats, decreased L-[U-$^{14}$C]glutamine oxidation, but not D-[U- $^{14}$C]glucose oxidation in mesenteric lymphocytes of animals at 7 weeks of age, before the onset of insulitis.[11a] From these studies it was proposed that a remodelling of nutrient catabolism in MLN lymphocytes was a key event in the process by which this dietary manipulation affects autoimmune diabetes.

The purpose of the present study was to investigate further a possible association among diet, gut immune cells and islet endocrine tissue. To do this we investigated whether the same HC diet also affects L-[U-$^{14}$C]glutamine oxidation in isolated pancreatic islets and Peyer's patch cells and determined its effects on plasma D-glucose and insulin concentrations, pancreatic islet protein and insulin content, production of the suspected $\beta$-cell cytotoxin NO, by cultured islets, as well as mitogenic and biosynthetic activity in mesenteric lymphocytes in diabetes-prone and control, non-diabetes-prone BB rats.

### MATERIALS AND METHODS

The NIH-07 (NIH) diet is a standard, mainly cereal-based mixture composed of 82.5% plant materials which is associated with high diabetes frequency in BBdp rats. It includes dried skim milk (5%), fish meal (10%), soybean meal (12%), alfalfa meal (4%), corn gluten meal (3%), ground corn (24.5%), ground hard winter wheat (23%), wheat middlings (10%), Brewer's yeast (2%), molasses (1.5%), soybean oil (2.5%), plus minerals and vitamins. The hydrolyzed casein (HC) diet inhibits the development of diabetes and is a modification of the AIN-93G[12] diet containing 20% hydrolyzed casein (Red Star Bio-Products, Tara, Ontario) 51% corn starch, 12% sucrose, 7% soya oil, 5% cellulose-type fiber (Solka-Floc), supplemented with 3.5% AIN-93G mineral mix and 1.0% AIN-93G vitamin mix (ICN Biochemicals, Cleveland, OH, USA), 0.2% choline bitartrate, and 0.3% L-cystine.

Male and female diabetes-prone BB (BBdp) rats and control non-diabetes prone BB (BBc) rats were obtained from the colonies maintained at the Animal Resources Division, Health Canada, Ottawa and transferred to Brussels. Up to the day after their arrival in Brussels, they had access to the standard rodent diet, NIH. They were then weighed and housed in groups of 2–4 rats of the same sex in separate cages with free access to tap water and either powdered NIH diet or HC diet. Approximately 17 days later, the animals were again weighed and killed by decapitation. Blood was collected in heparinized tubes for the measurement of plasma D-glucose[13] and insulin[14] concentrations.

A single batch of about 600 islets was prepared by the collagenase procedure[15] from 4–5 rats of identical diabetes susceptibility and dietary status. From each batch, two groups of 15 islets each were sonicated in 250 µl of $H_2O$ for measurement of protein and insulin content.[15,16] Eight further groups of 15 islets each were incubated for 120 min at 37°C in 50 µl of a Hepes- and bicarbonate-buffered medium[17] equilibrated in a mixture of $O_2-CO_2$ (95–5, v–v) and containing bovine serum albumin (5 mg/ml) and L-[U-$^{14}$C]glutamine (1.0 mmol/l), for measurement of $^{14}CO_2$ production.[17]

Finally, 4 groups of 100 islets each were cultured in a microwell plate (Nunc, Roksilde, Denmark) for 48 hours at 37°C in 250 µl of RPMI 1640 medium (Gibco, Grand Island, New York, USA) supplemented with 10% fetal bovine serum, 100 U/ml penicillin, 100 µg/ml streptomycin, 2 mmol/l L-glutamine and, when required, 25 U/ml human recombinant IL-1$\beta$ (Genzyme, Cambridge, MA, USA). This medium was equilibrated in humidified air–$CO_2$ (95–5, v–v). After culture, the islets were collected in Eppendorf tubes, washed twice in cold phosphate buffer saline, and sonicated (3 × 10 s) in 1.0 ml of distilled water for measurement of protein and insulin.[15,16] The nitrite content of the culture medium was measured using a microplate assay.[18] Two aliquots (100 µl each) of the culture medium were mixed with 10 µl of Griess reagent. After 10 min incubation at 20°C, the absorbance was read at 540 nm in a microplate reader (Titertek Multiscan MCC/340 MKII Blab, Finland). Nitrite concentration was calculated using $NaNO_2$ as standard. This range of concentrations (50–500 pmol/sample) yielded a linear dose-response relationship, with mean readings derived from 4 individual experiments of 11.6 ± 0.9 and 11.2 ± 0.5 arbitrary units per pmol of nitrite with the lowest (50 pmol) and highest (500 pmol) standard amounts of $NaNO_2$, respectively. In pilot experiments conducted in islets from normal female Wistar rats, the production of NO during the first 24 hours of culture was increased ($p < 0.001$) by IL-1$\beta$ from a basal value of 0.33 ± 0.07 to 2.20 ± 0.15 pmol/islet per day ($n = 6-7$). In relative terms, the increase in NO production due to IL-1$\beta$ was less marked over 2–3 days of culture than after only 24 hours, not exceeding 77.1 ± 12.8% ($n = 4$; $p < 0.01$) of the mean corresponding basal value. The production of NO was proportional to the number of cultured islets (100–200 islets/sample), the measurements made in groups of 200 islets each averaging, when expressed per islet, 93.2 ± 10.8% ($n = 4$) of those made in smaller groups of only 100 islets each.

Peyer's patches from the same 4–5 rats were removed and passed through a 50-mesh grid in cold RPMI 1640 (Gibco) under sterile conditions. Cells were washed twice with RPMI 1640 and incubated in groups of $2 \times 10^5$ cells each for 120 min at 37°C in 0.1 ml of Hepes- and bicarbonate-buffered medium[17] containing bovine serum albumin (5 mg/ml), L-[U-$^{14}$C]glutamine (1.0 mmol/l), penicillin (250 U/ml) and streptomycin (250 µg/ml) for measurement of $^{14}CO_2$ production.[17]

Mesenteric lymph nodes from the same 4–5 rats were also passed through a 50-mesh grid in cold RPMI 1640 under sterile conditions. The cells were washed twice with RPMI 1640, and then cultured in a 96-well plate ($5 \times 10^5$ cells/well) for 48 hours at 37°C in 200 µl of RPMI 1640 supplemented with 2.5 µmol/l 2-mercaptoethanol, 10% heat-inactivated fetal bovine serum, 100 U/ml penicillin, 100 µg/ml streptomycin, 2 mmol/1 L-glutamine and, when required 5 µg/ml concanavalin A (Sigma Chemical, St. Louis, MO, USA). This medium was equilibrated with air–$CO_2$ (95–5, v–v). Cells were pulsed with [methyl-$^3$H]thymidine (1 μCi/well; NEN Products, Boston, MA, USA) for the last 18 hours of culture, and then harvested onto glass fiber filters using an automated cell harvester (Packard Instrument Co., Rockville, MD, USA). After drying, the filters were immersed in universal Microscint cocktail (Packard) and radioactivity was measured using an automated microplate scintillation counter (Packard).

Other groups of $2 \times 10^5$ mesenteric lymph node cells each were incubated for 120 min at 37°C in 80 μl of a bicarbonate-buffered medium[15] containing 5 mg/ml bovine serum albumin, 7.0 mmol/l D-glucose and 37 μmol/l L-[4-$^3$H]phenylalanine (0.1 mCi/ml). The cells were then washed twice with the same buffer containing 1.0 mmol/l unlabelled L-phenylalanine, and tritiated TCA-precipitable and TCA-soluble material were measured.[19]

All results are presented as means ± SE, together with the number of individual determinations ($n$). The statistical significance of differences between mean values was assessed by Student's two-tailed $t$-test.

### RESULTS

### Metabolic and Hormonal Status

The sex ratio, age, body weight, duration of diet administration, daily weight gain and plasma D-glucose concentration were all comparable in control and BBdp rats, regardless of diet treatment (Tab. I). The plasma insulin concentration and plasma insulin/glucose paired ratio were lower, however, in BBdp rats than in BBc animals fed NIH ($p < 0.005$). The HC diet increased the plasma insulin concentration in BBdp rats ($p < 0.02$), but not in control animals ($p > 0.2$).

### Pancreatic Islets

Protein content of the islets was lower in BBdp rats *versus* control rats (240 ± 22 ($n = 47$) *vs.* 409 ± 44 ng/islet ($n = 40$); $p < 0.001$). It appeared somewhat higher in the control animals fed the HC diet ($p < 0.01$) but no diet effect was seen in BBdp rats ($p > 0.2$; Tab. II).

In freshly isolated islets, the insulin content, expressed per islet, was lower ($p < 0.005$) in BBdp than in control rats, regardless of diet. The lowest insulin content was observed in BBdp rats fed NIH. The HC diet increased ($p < 0.02$) the insulin content in BBdp rats, but not in control animals. The insulin content tended to be lower in islets cultured for 2 days than in freshly isolated islets, but this difference was only statistically significant in BBdp rats ($p < 0.05$). In cultured islets, the insulin content was also lower ($p < 0.001$) in BBdp rats than in control animals, regardless of diet ($p < 0.001$). Cultured islets from both BBc and BBdp rats fed the HC diet had increased ($p < 0.005$) insulin content.

In freshly isolated islets, the paired ratio between insulin and protein content was also lower ($p < 0.001$) in BBdp rats (0.68 ± 0.11 mU/μg; $n = 8$) than in BBc animals (1.29 ± 0.07 mU/μg; $n = 8$) when both were fed the NIH diet. In animals fed HC, there was a decrease ($p < 0.05$) of this ratio in control rats to 0.94 ± 0.12 mU/μg ($n = 8$), whereas the ratio was increased ($p < 0.005$) in BBdp rats to 1.23 ± 0.09 mU/μg ($n = 8$). As a result, these two mean values were no longer significantly different.

Similarly, in cultured islets, the paired ratio between insulin and protein content was lower ($p < 0.001$) in BBdp rats (0.88 ± 0.12 mU/μg; $n = 16$) than in BBc animals (1.93 ± 0.10 mU/μg; $n = 16$) fed NIH. In the BBdp animals the ratio of insulin/protein was increased ($p < 0.001$) by feeding the HC diet to 1.92 ± 0.18 mU/μg ($n = 15$). In this series of measurements, the HC diet also increased the insulin/protein ratio in BBc rats. Pooling the results obtained in freshly isolated and cultured islets, the paired ratios between insulin and protein content in the control rats fed the NIH diet and the HC diet respectively were 1.72 ± 0.09 ($n = 24$) and 1.87 ± 0.26 ($n = 16$) mU/μg.

TABLE I Metabolic and hormonal status

| Rats | Control, BBc | | Diabetes-prone, BBdp | |
|---|---|---|---|---|
| diet | NIH | HC | NIH | HC |
| Sex (M/F) | 8/8 | 8/8 | 9/8 | 7/10 |
| Age (d) | 51.9 ± 0.3 (16) | 53.0 ± 0.9 (16) | 48.2 ± 0.5 (17) | 49.4 ± 0.3 (17) |
| Body wt. (g) | 179 ± 10 (16) | 175 ± 7 (16) | 160 ± 5 (17) | 172 ± 6 (17) |
| Duration of treatment (d) | 18.5 ± 0.4 (16) | 18.5 ± 0.1 (16) | 15.1 ± 0.5 (17) | 14.9 ± 0.3 (17) |
| Weight gain during treatment (g/d) | 5.5 ± 0.4 (16) | 5.2 ± 0.3 (16) | 5.5 ± 0.3 (17) | 5.6 ± 0.3 (17) |
| Plasma glucose (mmol/l) | 10.21 ± 0.19 (15) | 10.10 ± 0.25 (15) | 9.55 ± 0.19 (17) | 10.62 ± 0.22 (17) |
| Plasma insulin (μU/ml) | 44.2 ± 4.4 (15) | 52.5 ± 6.0 (13) | 25.5 ± 1.8 (17)[a,b] | 32.9 ± 2.1 (17)[a,b] |
| Plasma insulin/glucose ratio (U/mol) | 4.37 ± 0.47 (15) | 5.43 ± 0.52 (12) | 2.66 ± 0.18 (17)[a,b] | 3.10 ± 0.18 (17)[a,b] |

[a] $p < 0.005$, Both values significant compared with BBc.
[b] $p < 0.02$, BBdp, NIH *vs.* HC.

TABLE II Pancreatic islets

| Rats | Control, BBc | | Diabetes-prone, BBdp | |
|---|---|---|---|---|
| diet | NIH | HC | NIH | HC |
| Protein content (ng/islet) | 314 ± 33 (24)[a] | 552 ± 88 (16)[a] | 266 ± 35 (24) | 213 ± 25 (23) |
| Insulin content (μU/islet) | | | | |
| – freshly isolated islets | 615 ± 73 (8) | 723 ± 63 (8) | 255 ± 34 (8)[b] | 423 ± 49 (8)[b] |
| – cultured islets | 456 ± 47 (16)[b] | 808 ± 39 (16)[b] | 162 ± 21 (16)[c] | 308 ± 45 (14)[c] |
| L-[U-$^{14}$C]glutamine oxidation (pmol/islet per 120 min) | 58.3 ± 4.0 (32) | 54.4 ± 4.3 (32) | 62.0 ± 4.2 (32)[d] | 48.4 ± 3.3 (32)[d] |
| NO production (pmol/islet per 48 hours) | | | | |
| – basal | 6.2 ± 0.6 (8)[e] | 6.8 ± 0.9 (8) | 2.5 ± 0.6 (8)[e] | 4.3 ± 0.9 (8) |
| – stimulated with IL-1$\beta$ | 8.4 ± 0.3 (8) | 8.3 ± 0.6 (8) | 8.8 ± 0.5 (8) | 9.1 ± 0.3 (7) |
| – stimulated/basal (%) | 140 ± 2 (8)[f] | 127 ± 5 (8)[f] | 424 ± 21 (8)[g] | 243 ± 6 (7)[g] |
| NO production (pmol/μg protein per 48 hours) | | | | |
| – basal | 30.7 ± 6.7 (8) | 33.9 ± 4.3 (4) | 14.4 ± 3.7 (8)[h] | 33.4 ± 7.9 (8)[h] |
| – stimulated with IL-1$\beta$ | 40.5 ± 5.5 (8) | 36.0 ± 3.4 (4) | 56.2 ± 7.4 (8) | 73.3 ± 15.7 (6) |

[a] $p < 0.01$, BBc, NIH *vs.* HC.
[b] $p < 0.02$, Both BBc and BBdp, NIH *vs.* HC.
[c] $p < 0.02$, Both BBc and BBdp, NIH *vs.* HC.
[d] $p < 0.02$, BBdp, NIH *vs.* HC.
[e] $p < 0.001$, BBc *vs.* BBdp, both fed NIH.
[f] $p < 0.05$, BBc, NIH *vs.* HC.
[g] $p < 0.001$, BBdp, NIH *vs.* HC.
[h] $p < 0.05$, NIH *vs.* HC.

When expressed per islet, the oxidation of L-[U-$^{14}$C]glutamine (1.0 mmol/l), which was measured over 120 min incubation in the absence of any other exogenous nutrient, was not significantly different in BBc and BBdp rats fed the NIH diet. Relative to these reference values, the HC diet did not affect significantly the oxidation of L-[U-$^{14}$C]glutamine in control animals, but decreased it in BBdp rats ($p < 0.02$, Tab. II).

Expressed per islet, the basal production of NO over 2 days of culture was higher ($P < 0.001$) in control compared with diabetes-prone BB rats fed the NIH diet. The HC diet did not affect significantly the basal production of NO in control rats. In the BBdp rats, however, the mean values for basal NO production were higher in the animals fed the HC diet. This difference was significant when the production of NO was expressed relative to the protein content of the islets ($P < 0.05$). In considering these comparisons, it should be recalled that the production of NO may be affected by the contribution of distinct endocrine, as well as non-endocrine cell types relative to the total islet mass.

In all cases, IL-1$\beta$ increased NO production. In relative terms, the enhancing action of the cytokine was more pronounced in diabetes-prone than control rats, regardless of diet. Feeding the HC diet decreased the enhancing action of IL-1$\beta$ in both BBc and BBdp rats. This effect was most obvious in BBdp animals, however, the HC diet failed to restore responsiveness to the cytokine to the lower level found in control animals ($P < 0.001$).

### Peyer's Patch Cells

The oxidation of L-[U-$^{14}$C]glutamine (1.0 mmol/l) by Peyer's patch cells incubated in the absence of any other exogenous nutrient was not significantly different in BBc rats and BBdp animals fed the NIH diet (Tab. III). In the control rats fed HC, L-[U-$^{14}$C]glutamine oxidation was decreased ($p < 0.001$), in a manner similar to that previously documented in mesenteric lymphocytes.[11a] In the BBdp animals, however, the HC diet markedly increased the oxidation of the amino acid ($p < 0.001$).

### Mesenteric Lymph Node Cells

The basal incorporation of tritiated thymidine in mesenteric lymphocytes was about 3 times higher ($p < 0.001$) in BBdp rats than in control animals when both were fed the NIH diet (Tab. III). It was slightly increased ($P < 0.001$) by the HC diet in control animals, but markedly decreased ($p < 0.001$) by this diet in the BBdp rats. Concanavalin A strikingly increased thymidine incorporation in all cases. In the presence of concanavalin A, the incorporation of thymidine was still slightly higher ($p < 0.001$) in BBdp than BBc rats fed NIH diet. This was not the case, however, in animals fed the HC diet. Indeed, the HC diet slightly increased concanavalin A-stimulated thymidine incorporation in lymphocytes from control rats, but slightly decreased it in lymphocytes from BBdp animals ($p < 0.02$).

The effects of diet on basal thymidine incorporation in BBdp rats were, as a rule, paralleled by comparable changes in the biosynthetic activity of the mesenteric lymphocytes. Thus,

TABLE III Peyer's patch and mesenteric lymph node cells

| Rats | Control, BBc | | Diabetes-prone, BBdp | |
|---|---|---|---|---|
| diet | NIH | HC | NIH | HC |
| Peyer's patch cells | | | | |
| L-[U-$^{14}$C]glutamine oxidation (fmol/10$^3$ cells per 120 min) | 658 ± 50 (32)[a] | 340 ± 17 (32)[a] | 598 ± 74 (32)[b] | 1013 ± 63 (32)[b] |
| Mesenteric lymphocytes | | | | |
| [Methyl-$^3$H]thymidine incorporation (cpm/500 cells) | | | | |
| -basal | 2.5 ± 0.1 (40)[a] | 3.7 ± 0.2 (40)[a] | 8.4 ± 0.8 (40)[b] | 4.8 ± 0.4 (40)[b] |
| -concanavalin A | 298.6 ± 6.9 (40)[c] | 324.9 ± 3.3 (40)[c] | 342.0 ± 8.0 (40)[d] | 314.3 ± 7.4 (40)[d] |
| L-[4-$^3$H]phenylalanine incorporation (fmol/10$^3$ cells per 120 min) | | | | |
| -TCA-precipitable material | 1.31 ± 0.22 (37)[e] | 2.16 ± 0.22 (40)[e] | 2.44 ± 0.18 (39) | 2.34 ± 0.23 (37) |
| -TCA soluble material | 0.09 ± 0.01 (37) | 0.04 ± 0.01 (40) | 0.06 ± 0.01 (39) | 0.04 ± 0.01 (37) |

[a] $p < 0.001$, BBc, NIH vs. HC.

[b] $p < 0.001$, BBdp, NIH vs. HC.

[c] $p < 0.02$, BBc, NIH vs. HC.

[d] $p < 0.02$, BBdp, NIH vs. HC.

[e] $p < 0.01$, BBc, NIH vs. HC.

the incorporation of L-[4-$^3$H]phenylalanine into TCA-precipitable material over 120 min incubation at 7.0 mmol/l D-glucose in BBdp rats was about twice that ($p < 0.001$) of BBc rats (both fed the NIH diet, Tab. III). Phenylalanine incorporation was increased ($p < 0.01$) in control animals fed the HC diet, but was not significantly affected by this diet in BBdp rats. The HC diet decreased ($p < 0.001$) the pool of tritiated TCA-soluble material from $85.9 \pm 9.4$ to $42.3 \pm 3.0$ amol/$10^3$ cells in control rats, and from $60.4 \pm 2.7$ to $39.9 \pm 3.5$ amol/$10^3$ cells in BBdp animals ($n = 37-40$).

### DISCUSSION

Dietary modification of autoimmune diabetes in BB rats is a complex interaction involving the source of dietary amino acids and several predisposing factors that coalesce in a destructive process focused on the pancreatic islets.[1] This interaction begins as early as weaning, before classic insulitis is observed.[1,7,8] The present results provide further evidence in support of the suggestion that dietary manipulation of diabetes expression involves multiple sites.[1,8,11] In the present study, feeding diabetes-prone BB rats a protective HC diet affected the target islet tissue and cells from the mesenteric lymph nodes and Peyer's patches in keeping with a previous report.[11a] The protective diet also affected basal and IL-1$\beta$ stimulated islet NO production and enhanced islet insulin content, providing additional evidence[1,8] that beta cells may participate in their own destruction,[20,21] and further highlighting an important food modifiable interaction between the gut and islets in diabetes pathogenesis.

In diabetes-prone animals, feeding the HC diet increased plasma insulin concentration and islet insulin content, without increasing islet protein content. It also increased the basal production of NO by pancreatic islets and the oxidation of L-[U-$^{14}$C]glutamine by Peyer's patch cells, while decreasing L-[U-$^{14}$C]glutamine oxidation in the islets and basal mitotic activity in mesenteric lymphocytes.

These findings lead to the following speculative proposals. Peyer's patch cells could be among the first cells affected by the pathogenic process leading to autoimmune diabetes in BB rats. Although oxidation of L-[U-$^{14}$C]glutamine by these cells was not significantly different in control and diabetes-prone rats fed the NIH diet, it was modified in opposite directions by feeding the HC diet to control and BBdp animals. This clearly indicates that the nutritional, and presumably functional, status of these cells is affected by dietary factors in a different manner in control and diabetes-prone BB rats.

Mesenteric lymph node cells also appear to be participating in the autoimmune process in BB rats. In addition to the metabolic changes recently documented in these cells,[11a] the mitotic and biosynthetic activities were also affected by rat type and diet. Although the differences seen when comparing BBc with BBdp MLN may reflect in part the lymphopenia that occurs in BBdp animals, diet effects in these rats have not yet been linked directly to this trait. The increased basal mitotic and biosynthetic activities found in mesenteric lymphocytes of BBdp *versus* control rats fed NIH diet are consistent with activation of these cells in BBdp animals. The dietary treatment had different effects depending on rat type. Feeding the HC diet augmented both variables in control rats, but decreased basal incorporation of tritiated thymidine in MLN cells from BBdp rats.

Pancreatic islets are currently considered to be the main target of this autoimmune process. An important effect was the enhancement of islet insulin content in BBdp rats fed the HC diet. Islet insulin/protein content increased from $0.88 \pm 0.12$ in NIH-fed to $1.92 \pm 0.18$ mU/µg in HC-fed BBdp rats while plasma insulin concentration and insulinogenic index also increased by feeding the HC diet to BBdp rats, but not control rats. This is consistent with our previous report of enhanced islet area in HC-fed BBdp rats[8] and recent reports that NOD mice have decreased islet mass before disease onset.[27]

Basal production of NO was lower in pancreatic islets of diabetes-prone compared with control rats fed the NIH diet. This difference could not be accounted for solely by the higher protein content of the islets from control rats fed the NIH diet. Because of the low basal NO levels, the extent of the IL-1$\beta$-induced increase in NO production was higher in BBdp rats than in controls. Moreover, in HC-fed BBdp rats, both the basal production of NO and IL-1$\beta$ stimulated NO production approached control values and were no longer significantly different from control rats fed the NIH diet (Tab. II). The current results are also consistent with those of Bone *et al.*, who reported a unique BB rat subpopulation, BB/S-R that was resistant to developing diabetes.[24] These animals also showed a lack of IL-1$\beta$ stimulation of NO production in isolated islets when compared with control Wistar rats. A possible link to the metabolism of the NOS substrate, arginine, in the islets must also be considered as it has been reported that arginine metabolism is impaired in BBdp rat coronary endothelial cells.[25] In addition, resting BBdp islet endothelial cells in culture show decreased NO production compared with cells from diabetes resistant BB and Wistar rats, a result of abnormally low activity of endothelial constitutive nitric oxide synthases (*ec*NOS, 26). The present findings also show that basal and IL-1$\beta$-stimulated NO production in the pancreatic islets of BBdp rats is abnormal and this defect is partially restored in animals fed a protective HC diet.

Activated macrophages, capable of producing IL-1$\beta$, are the first inflammatory cells to enter the pancreas in BBdp rats.[22] In addition, the target $\beta$-cells may be stimulated to express inducible nitric oxide synthase (iNOS) by IL-1$\beta$, resulting in increased production of NO, inhibition of mitochondrial glucose oxidation, damage to various enzymes and inhibition of insulin secretion.[23] Recently, it was proposed that in addition to a role as a cytotoxic mediator of $\beta$-cell destruction, NO may act as an important immunoregulator of Th1/Th2 balance, down-regulating Th1 cytokines such as IL-2 and IFN$\gamma$ and increasing Th2-associated compounds leading to a Th2 predominance.[22] The present results are consistent with this suggestion as the protective HC diet, which leads to a predominance of Th2/Th3 cytokines over Th1 in the pancreas,[8] increased pancreatic NO levels close to those seen in BBc rats.

The results from the present studies show that in BBdp rats fed HC, the islets have increased insulin content, decreased glutamine oxidation, and basal NO production (μmol/μg protein per 48 h) is similar to levels seen in control rats (Tab. II). The capacity of IL-1$\beta$ to stimulate NO production was dampened in BBdp animals fed HC. In addition, the HC diet had effects on MLN cells suggesting decreased activation as indicated by decreased glutamine oxidation and reduced basal and con A stimulated thymidine incorporation. Therefore, islets and MLN cells from HC-fed BBdp rats were metabolically down-regulated and this could explain in part how a majority of animals fed this diet is protected from developing diabetes. An interesting but as yet unexplained finding was that glutamine oxidation in PP cells was enhanced in BBdp rats fed HC while the converse was seen in BBc rats.

In conclusion, the present findings draw attention to differences in metabolic, mitotic, biosynthetic and hormonal variables between control and diabetes-prone BB rats at three cellular levels: Peyer's patch cells, MLN cells and pancreatic islets, all of which were influenced by a dietary manipulation that inhibits diabetogenesis. There may be reciprocal effects in HC-fed BBdp rats as reflected by enhanced glutamine oxidation by PP cells at the same time when activity of the islets was decreasing and responsiveness to IL-1$\beta$ was blunted. These studies further emphasize that the protective effect of feeding an HC diet on development of spontaneous diabetes in these animals is inextricably linked to changes in the target islet cells and to selected cells of the gut immune system.

### Acknowledgments

This study was supported in part by a grant from the Medical Research Council of Canada to FWS (MT-14125), Health Canada and a grant from the Belgian Foundation for Scientific Medical Research to WJM (3.4513.94). The technical assistance of H. Gruber, J. Souligny of Health Canada in Ottawa and J. Schoonheydt, M. Urbain and the secretarial help of C. Demesmaeker in Brussels are gratefully acknowledged.

### References

[1] Scott, F. W. (1996). Food-induced type 1 diabetes in BB rats. *Diabetes/Metabolism Reviews*, **12**, 341–359.

[2] Coleman, D. L., Kuzawa, J. E. and Leiter, E. H. (1990). Effect of diet on incidence of diabetes in nonobese diabetic mice. *Diabetes*, **39**, 432–436.

[3] Karges, W., Hammond-McKibben, D., Cheung, R. K., Visconti, M., Shibuya, N., Kemp, D. and Dosch, H. M. (1997). Immunological aspects of nutritional diabetes prevention in NOD mice: a pilot study for the cow's milk-based IDDM prevention trial. *Diabetes*, **46**, 557–64.

[4] Scott, F. W., Norris, J. M. and Kolb, H. (1996). Milk and type I diabetes: examining the evidence and broadening the focus. *Diabetes Care*, **19**, 379–383.

[5] Akerblom, H. A. and Knip, M. (1998). Putative environmental factors in type 1 diabetes. *Diabetes/ Metabolism Reviews*, **14**, 31–67.

[6] Scott, F. W. (1994). Food, diabetes and immunology. In: *Diet, Nutrition and Immunity*, Forse, R. A., Bell, S. J., Blackburn, G. L. and Kabbash, L. G. (Eds.), CRC Press, Boca Raton, FL, pp. 71–92.

[7] Li, X. B., Scott, F. W., Park, Y. H. and Yoon, J. -W. (1995). Low incidence of autoimmune type I diabetes in BB rats fed a hydrolysed casein-based diet associated with early inhibition of non-macrophage-dependent hyperexpression of MHC class I molecules on beta cells. *Diabetologia*, **38**, 1138–1147.

[8] Scott, F. W., Cloutier, H. E., Kleemann, R., Wörz-Pagenstert, U., Modler, H. W. and Kolb, H. (1997). Potential mechanisms by which certain foods promote or inhibit the development of spontaneous diabetes in BB rats: dose, timing, early effect on islet area and switch in infiltrate from Th1 to Th2 cells. *Diabetes*, **46**, 589–598.

[9] Kolb, H. (1997). Benign *versus* destructive insulitis. *Diabetes Metab. Rev.*, **13**, 139–146.

[10] Bellmann, K., Kolb, H., Hartmann, B., Rothe, H., Rowsell, P., Rastegar, S., Burghardt, K. and Scott, F. W. (1997). Intervention in autoimmune diabetes by targeting the gut immune system. *Int. J. Immunopharmacol.*, **19**, 573–577.

[11] Scott, F. W. (1988). Dietary initiators and modifiers of BB rat diabetes: a summary and working hypothesis. In: *Lessons From Animal Diabetes II, 2nd International Workshop*, Eds. Shafrir, E. and Renold, A. E., Pub., John Libbey & Company Ltd., London. pp. 34–39.
(a) Scott, F. W., Olivares, E., Sener, A., Malaisse, W. J., Dietary effects on insulin and nutrient metabolism in mesenteric lymph node cells, splenocytes and pancreatic islets of BB rats. *Metabolism* (in press) .

[12] Reeves, P. G., Nielsen, F. H. and Fahey, G. C. (1993). AIN-93 purified diets for laboratory rodents: final report of the American Institute of Nutrition ad hoc writing committee on the reformulation of the AIN-76A rodent diet. *J. Nutr.*, **123**, 1939–1951.

[13] Bergmeyer, H. U. and Berndt, E. (1974). Measurement of glucose with glucose oxidase and peroxidase. In: Bergmeyer, H. U. (Ed.), *Methods of Enzymatic Analysis*, Academic Press, New York, pp. 1205–1215.

[14] Leclercq-Meyer, V., Marchand, J., Woussen-Colle, M.-C., Giroix, M.-H. and Malaisse, W. J. (1985). Multiple effects of leucine on glucagon, insulin and somatostatin secretion from the perfused rat pancreas. *Endocrinology*, **116**, 1168–1174.

[15] Malaisse-Lagae, F. and Malaisse, W. J. (1984). Insulin release by pancreatic islets. In: Larner, J. and Pohl, S. L. (Eds.), *Methods in Diabetes Research*. John Wiley & Sons, New York, pp. 147–152.

[16] Lowry, O. H., Rosebrough, N. H., Farr, A. and Randall, R. J. (1951). Protein measurement with the Folin phenol reagent. *J. Biol. Chem.*, **193**, 265–275.

[17] Malaisse, W. J. and Sener, A. (1988). Hexose metabolism in pancreatic islets. Feedback control of D-glucose oxidation by functional events. *Biochim. Biophys. Acta*, **971**, 246–254.

[18] Ding, A. H., Nathan, C. F. and Stuehr, D. J. (1988). Release of nitrogen intermediates and reactive oxygen intermediates from mouse peritoneal macrophages. *J. Immunol.*, **141**, 2407–2412.

[19] Laghmich, A., Ladrière, L., Malaisse-Lagae, F., Dannacher, H., Björkling, F. and Malaisse, W. J. (1998). Stimulation of biosynthetic activity by novel esters in rat pancreatic islets. *Biochem. Pharmacol.*, **55**, 909–913.

[20] Homo-Delarche, F. and Boitard, C. (1996). Autoimmune diabetes: the role of the islets of Langerhans. *Immunology Today*, **17**, 456–460.

[21] Gotfredsen, C. F., Buschard, K. and Frandsen, K. (1985). Reduction of diabetes incidence of BB Wistar rats by early prophylactic insulin treatment of diabetes-prone animals. *Diabetologia*, **28**, 933–935.

[22] Kolb, H. and Kolb-Bachofen, V. (1998). Nitric oxide in autoimmune disease: cytotoxic or regulatory mediator? *Immunology Today*, **19**, 556–561.

[23] Nerup, J. (1994). On the pathogenesis of IDDM. *Diabetologia*, **37**, [Suppl 2], S82–S89.

[24] Bone, A. J., Hitchcock, P. R., Gwilliam, D. J., Cunningham, J. M. and Barley, J. (1999). Insulitis and mechanisms of disease resistance: studies in an animal model of insulin dependent diabetes mellitus. *J. Mol. Med.*, **77**, 57-61.

[25] Wu, G. and Meininger, C. J. (1995). Impaired arginine metabolism and NO synthesis in coronary endothelial cells of the spontaneously diabetic BB rat, *Am. J. Physiol*, **269**, H1312-1318.

[26] Suschek, C. V., Bonmann, E. and Kolb-Bachofen, V. (1999). A regulatory defect of constitutive NO-synthase in islet endothelial cells correlates with probability of disease manifestation in BBdp rats. *Diabetologia*, **42**, 457-464.

[27] Sreenan, S., Pick, A. J., Levisetti, M., Baldwin, A. C., Pugh, W. and Polonsky, K. S. (1999). Increased beta-cell proliferation and reduced mass before diabetes onset in the nonobese diabetic mouse. *Diabetes*, **48**, 989-96.

